# NET burden in left atrial blood is associated with biomarkers of thrombosis and cardiac injury in patients with enlarged left atria

**DOI:** 10.1007/s00392-024-02464-9

**Published:** 2024-06-26

**Authors:** Kimberly Martinod, Annika Claessen, Caroline Martens, Krystin Krauel, Leydi Carolina Velásquez Pereira, Jens Witsch, Thilo Witsch

**Affiliations:** 1 Center for Molecular and Vascular Biology, Department of Cardiovascular Sciences, KU Leuven, Leuven, Belgium; 2 Department of Cardiology and Angiology, Faculty of Medicine, University Heart Center Freiburg-Bad Krozingen, University of Freiburg, Hugstetter Str 55, 79106 Freiburg, Germany; 3 Department of Neurology, Hospital of the University of Pennsylvania, Philadelphia, PA USA; 4 Present Address: Department of Cardiology, Angiology, Haemostaseology and Medical Intensive Care, University Medical Center Mannheim, Medical Faculty Mannheim, Heidelberg University, Mannheim, Germany

**Keywords:** Atrial fibrillation, Thromboinflammation, Neutrophil extracellular traps, Von Willebrand factor

## Abstract

**Background:**

Emerging data suggest an association between left atrial (LA) enlargement, thrombus formation, and ischemic stroke. However, it is unknown what may mediate such clot formation in LA dysfunction. Neutrophils promote large vessel occlusion and microthrombosis via neutrophil extracellular trap (NET) release, thus lying at the interface of inflammation, thrombosis, and fibrosis.

**Approach:**

We conducted a prospective all-comers cohort study in patients undergoing catheterization procedures with atrial transseptal access (MitraClip, MC; left atrial appendage closure, LAAC; pulmonary vein ablation, PVA; patent foramen ovale closure, PFO). We measured NETs, cytokines, thrombotic factors, and cardiac injury markers in paired blood samples collected from peripheral blood and within the left atrium. We correlated these biomarkers with echocardiographic measures of LA structure and function (including left atrial volume index, LAVI). Data were analyzed by procedure type, and stratified by LAVI or atrial fibrillation (AF) status.

**Results:**

We enrolled 70 patients (mean age 64 years, 53% women). NETs, but not other markers, were elevated in LA compared to peripheral blood samples. Most thrombotic, inflammatory, and cardiac damage markers were elevated in LAs from MC or LAAC compared to PFO patients. Overall, NET biomarkers positively correlated with VWF, LAVI, and markers of cardiac injury and negatively with ADAMTS13 activity. LA enlargement and the presence of AF similarly stratified patients based on thromboinflammation measurements, but this was not limited to AF at the time of sample collection.

**Conclusion:**

Elevated NETs and VWF in patients with enlarged LA or AF suggest enhanced thromboinflammation within the LA.

**Supplementary Information:**

The online version contains supplementary material available at 10.1007/s00392-024-02464-9.

## Introduction

Despite recent advances in stroke treatment, ischemic stroke is still the second leading cause of death worldwide as of Dec 2020 [18] (WHO Fact Sheet, The Top 10 Causes of Death). Twenty percent of embolic strokes are of undetermined etiology (ESUS). Emerging data suggest that left atrial cardiopathy is associated with clot formation in the left atrium that then leads to stroke even in the absence of atrial fibrillation (AF). Due to the lack of mechanistic insight into the stroke etiology in patients with ESUS there are no highly effective prevention strategies available and direct oral anticoagulants (DOAC), effective at reducing the risk of stroke in patients with AF, have been shown to be inferior to aspirin in two randomized controlled trials [4, 6]. It is therefore of interest to identify novel targets that could contribute to thrombus formation in the LA, and to better understand the local thromboinflammatory environment within the heart.

The formation of neutrophil extracellular traps (NETs) has been identified as a crucial mechanism in thrombotic diseases, including in the formation of cardioembolic thrombi. Moreover, emerging evidence suggests that von Willebrand factor (VWF) plays a vital role in NETs-mediated pathologies, serving as a bridge between endothelium and activated platelets/leukocytes. Both have also been shown to be involved in fibrotic remodeling of the heart [15, 25]. VWF and NETs have been described as acute-phase reactants in systemic inflammatory diseases including sepsis, cancer, and COVID-19 [1, 21]. NETs are extracellular chromatin strands released from activated or dying neutrophils, lined with granule proteins such as myeloperoxidase or neutrophil elastase [2]. They can directly promote myofibroblast differentiation in vitro and are found in the proximity of IL-17-producing cells which likely recruit additional neutrophils to the thrombus [3]. This is a potential druggable interaction to prevent inflammation-mediated fibrosis development, especially when microthrombosis exacerbated by NETs is involved in the disease etiology.

NETs have been reported in a wide variety of thrombus samples collected from human patients, including from acute ischemic stroke [5, 11], myocardial infarction [12], venous thromboembolism [19], and most recently from in situ thrombus formation in the pulmonary vasculature [16]. This can be identified using citrullinated histones, decondensed chromatin, or neutrophil granule enzymes colocalizing with DNA structures outside of the cell. In ischemic stroke, NET-rich thrombi were initially shown to be associated with stroke of cardioembolic origin [11], although this was not a reproducible finding in a later, larger study involving a larger number of collected endovascular thrombi [23].

Atrial cardiopathy is proposed to create thrombogenicity and enhance clot formation, even independently of atrial fibrillation [8]. Elevated NETs have recently been reported in systemic circulation in patients with enlarged left atria and atrial fibrillation [17]. The aim of this study was to identify biomarkers that correlate with structural abnormalities of the heart, with a focus on the left atrium. We hypothesized that blood within the heart, here specifically the LA, may have higher local concentrations of prothrombotic factors including NETs, so designed the study to obtain these samples during clinically necessary catheterization procedures. This may provide insight into pathomechanisms that drive thromboembolic risks rooted in atrial dysfunction.

## Methods

### Study design and patient population

In this prospective single-center study, adult patients scheduled for a procedure requiring atrial transseptal puncture were recruited at the cardiology wards of the Heart Center, University of Freiburg between July 29th, 2020, and February 18th, 2021. The local Institutional Review Board approved this study (file number 199/20: Ethics Committee of the University of Freiburg, Germany). Written informed consent was obtained from each participant prior to study inclusion.

Paired blood samples (peripheral venous and left atrial) were collected from patients undergoing catheterization with atrial transseptal puncture. These procedures included MitraClip placement (MC, *n* = 14), pulmonary vein ablation (PVA, *n* = 29), or left atrial appendage closure (LAAC, *n* = 3). In parallel, a group of patients were enrolled who underwent catheterization for a patent foramen ovale (PFO) or atrioseptal defect (ASD) closure and served as the control population (*n* = 24; *n* = 21 PFO, and *n* = 3 ASD). All patients received heparin as part of the catheterization procedure.

**Fig. 1:**
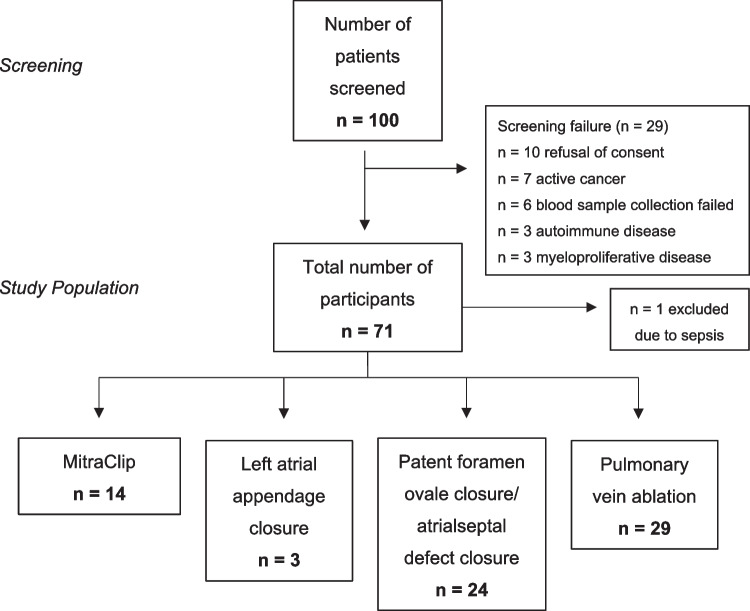
Flow chart of patient recruitment based on inclusion and exclusion criteria

Patients with infections, active malignancies, inflammatory diseases, or current thrombotic diseases were excluded. We also excluded participants with factors that may increase NET formation and that are not directly linked to thrombosis: active cancer, rheumatoid arthritis, systemic lupus erythematosus, previous pregnancy with preeclampsia, or other complications that have occurred. Lastly, we also excluded patients with known antiphospholipid syndrome, deep vein thrombosis, major surgery or physical trauma within the past 3 months, hereditary thrombophilia, or hemophilia. Patients with current pregnancy did not undergo elective cardiac procedures and were excluded from this study as well. Although not formally in the exclusion criteria at the time of recruitment, all patients were confirmed to be SARS-CoV-2 negative prior to the study as part of routine testing protocol during hospital intake. A flowchart of patient recruitment is provided in Fig. 1.

Follow-up was performed with telephone interviews to obtain information about thrombotic events, bleeding episodes, and anticoagulation received in the 1-year period after initial enrollment.

### Sampling and data collection

All clinical, imaging, and laboratory data was gathered prospectively and entered in case report forms by dedicated research staff. Both transthoracic and transesophageal echocardiography (TTE and TEE, respectively) were part of the patients’ routine preparation for the scheduled procedures. Echocardiographic images were assessed by a certified sonographer blinded to the remaining study data. Two blood samples were collected on average within 8 h of each other (longest within 24 h): one drawn from a peripheral vein before the procedure and one upon atrial transseptal catheter placement in the LA during the catheterization procedure. Atrial blood collection was performed slowly over 20 s through the catheter using no or only mild suction. Serum-separating, K2-EDTA, or 3.2% sodium citrate tubes were collected, and either immediately analyzed at the hospital’s in-house clinical laboratory (serum, EDTA), or used for plasma extraction (citrate) and later analyses.

### NET analysis

Platelet-poor plasma was prepared from freshly drawn citrate blood tubes within 1 h of blood collection. The blood was centrifuged at 800 g for 10 min at room temperature, followed by a second centrifugation of the supernatant at 3000 g for 7 min. Plasma was aliquoted and stored at − 80 °C until analysis. Plasma was diluted 1:2 for analysis of NET biomarkers (MPO-DNA complexes and citrullinated histone H3). Citrullinated histone H3 (H3Cit) was measured using the commercially available citrullinated histone H3 (Clone 11D3) ELISA kit (Cayman Chemical, catalog number 501620) following manufacturer instructions. MPO-DNA complexes were measured using an in-house ELISA modified from the Cell Death Detection ELISA (Roche #11,544,675,001). A 96-well MediSorp plate (ThermoFisher #467,320) was coated overnight at 4 °C with polyclonal anti-myeloperoxidase antibody (1:1000 dilution, ThermoFisher PA5-16,672 in 0.05 M sodium carbonate/sodium bicarbonate buffer (pH 9.6). After 4 washes with a wash buffer containing DPBS without Ca^2+^/Mg^2^ and 0.05% Tween-20, wells were blocked for 2 h at room temperature with incubation buffer from Cell Death Detection ELISA (Roche #11,544,675,001). Samples were diluted in that same incubation buffer and incubated for 1.5 h at room temperature on an orbital shaker at 50 rpm. After 4 washes, wells were incubated with mouse anti-DNA monoclonal antibody conjugated with peroxidase from the Roche Cell Death Detection ELISA for 1.5 h at room temperature on an orbital shaker at 50 rpm, washed 4 times, detected with ready-to-use TMB substrate (Life Technologies, #2023). The reaction was stopped with 1 N hydrochloric acid and the plate was then read at 450 nM with 630 nM background subtraction using a SpectraMax microplate reader. Values were normalized to a plasma pool calibrator from 18 healthy volunteers as multiple plates were needed to perform the full analysis. An in vitro standard prepared using the MPO standard from the LEGEND MAX™ Human Myeloperoxidase ELISA kit (Biolegend #440,007) incubated with native human nucleosomes (Merck Millipore, 14–1057) and lambda DNA from the Quant-iT™ PicoGreen™ dsDNA assay kit (Invitrogen #P7589) at known concentrations was used to generate a standard curve to calculate absolute concentrations, as previously reported [24].

### Further ELISA analyses

Peptidylarginine deiminase, type IV was measured using the PAD4 (human) ELISA Kit (Cayman Chemical, #501,460), soluble P-selectin was measured using the Human P-Selectin/CD62P Immunoassay (Quantikine ELISA, R&D Systems, #DPSE00), and platelet factor 4 (PF4) was measured using the Human PF4 SimpleStep ELISA Kit (CXCL4) (Abcam, #ab189573), following manufacturer instructions.

### Echocardiographic measurements

Cardiac dimensions (LA size, monoplane or biplane, left ventricular end-diastolic diameter), as well cardiac functions (left ventricular ejection fraction, E/E’) were measured at the echocardiography laboratories of the Heart Center, University of Freiburg, using standard transthoracic echocardiography tools. Flow velocity in the left atrial appendage was measured using pulse-wave Doppler during transesophageal echocardiography. The left atrial volume index was calculated using body surface area to correct for left atrial size measurements acquired in the biplane. For nine patients (2 PVA, 3 MC, 4 PFO), biplane LA size measurements were not available. For these patients, we estimated LAVI from the monoplane LA size measurement. A cutoff of 34 was used to distinguish normal from enlarged left atria, according to the most recent ESC guidelines [10].

### Statistical analysis

Descriptive statistics are presented as counts (*n*, %) for categorical variables, and mean (standard deviation, SD) or median (interquartile range, IQR) depending on the normality of data distribution. Due to majority non-normally distributed data (as determined by by Shapiro–Wilk tests), we performed all nonparametric analyses for comparisons among groups. For comparisons of continuous variables, we performed Mann–Whitney U tests for between-group comparisons and Wilcoxon matched-pairs signed rank tests for within-group paired comparisons. Statistical analyses were performed with a two-sided alternative hypothesis at the 5% significance level.

We conducted multivariable linear regression analyses to assess associations between LAVI and key thromboinflammatory markers. Covariates included in these models (age, neutrophils, VWF antigen, H3Cit) were selected based on the assumption—informed by the univariate association analysis and prior literature—that neutrophil and VWF parameters would both participate in a prothrombotic state and that both increase with increasing age. Age, neutrophil counts, VWF, H3Cit, and LAVI were all not normally distributed and therefore naturally log-transformed. The covariates included in the multivariable linear regression analysis were age, neutrophil levels, VWF antigen levels, and H3Cit. We ensured that the assumptions for linear regression, regarding linearity, homoscedasticity, and multicollinearity were met. Multicollinearity was defined as a variance inflation factor value of higher than 3.

Correlation matrices were made in R (version 4.1.1, R Foundation for Statistical Computing, R Core Team, Vienna, Austria) using non-parametric Spearman correlation coefficients. Correlograms were visualized using the corrplot package (version 0.9). No correction for multiple testing was performed. Heatmaps were created in R using the pheatmap package (version 1.0.12). Principal component analyses (PCA) were performed on an imputed dataset using the miss MDA package (version 1.18) given there were incomplete observations. PCA plots for individuals and biplots were made using the factoextra package (version 1.0.7). Multivariate analysis was performed using SPSS (version 29, IBM, Armonk, New York). The remaining statistical analyses and figures were made with GraphPad Prism version 9.5.0.

## Results

We prospectively included 70 patients in our study. 24 (34%) underwent PFO or ASD closure, 14 (20%) underwent MitraClip implantation, 29 (41.4%) underwent pulmonary vein ablation, and 3 (4.3%) underwent left atrial appendage closure. Baseline characteristics, routine clinical laboratory values, and medication use are presented in Table 1. Compared to the primary patient cohort, PFO/ASD closure patients were younger (mean age 52.3) and a majority (87.5%) had a prior stroke considered to be due to their PFO or ASD. PFO/ASD closure patients had a lower prevalence of diabetes and atrial fibrillation. There was no significant difference in CHA_2_DS_2_-VASc score, BMI, or in sex distribution between the groups. C-reactive protein (CRP), proBNP, and D-dimers were elevated in the primary cohort (MC, PVA, and LAAC) as compared to the control cohort (PFO). Patients in the PFO cohort received more aspirin therapy and patients undergoing PVA received more factor Xa inhibition.

**Table 1:** Patient characteristics and risk factors for stroke. Patients undergoing catheterization with atrial transseptal puncture access were recruited and classified according to their reason for undergoing the respective procedures. History of diabetes, stroke, and atrial fibrillation were noted, along with CHA_2_DS_2_-VASc score, standard clinical laboratory parameters, and medication use. Bold font indicates statistical significance. BMI, body mass index; CRP, C-reactive protein; DAPT, dual antiplatelet therapy; LAAC, left atrial appendage closure; proBNP, pro B-type natriuretic peptide

	Total	PFO/ASD Closure	MitraClip	Left atrial appendage closure	Pulmonary vein ablation	*p* values (PFO vs MC + LAAC)	*p* values (PFO vs PVA)	*p* values (MC + LAAC vs PVA)
*n*	70	24	14	3	29			
Clinical characteristics								
Age, y	63.9 ± 14 (22–92)	52.3	75.7	77.3	66.3	** < 0.0001**	**0.0003**	0.0585
Male	33 (47.1%)	12 (50%)	5 (35.7%)	3 (100%)	13 (44.8%)	> 0.9999	0.7858	> 0.9999
BMI (kg/m2)	28 ± 6.3	26.3	27.1	26.3	29.8	> 0.9999	0.1741	0.4348
Diabetes mellitus (%)	8 (11.4%)	1 (4%)	4 (28.6%)	1 (33.3%)	2 (6.9%)	0.0657	> 0.9999	0.0832
Prior stroke no. (%)	27 (38.6%)	21 (87.5%)	3 (21.4%)	0	3 (10.3%)	** < 0.0001**	** < 0.0001**	0.6552
Atrial fibrillation no. (%)	43 (61%)	0	11 (78.6%)	3 (100%)	29 (100%)	** < 0.0001**	** < 0.0001**	**0.0448**
No atrial fibrillation	27 (39%)	21 (87.5%)	3 (21.4%)	0	0	** < 0.0001**	** < 0.0001**	**0.0448**
Intermittent	27 (39%)	0	5 (35.7%)	2 (66.7%)	20 (69.0%)	**0.0009**	** < 0.0001**	**0.0004**
Permanent	16 (23%)	0	6 (42.9%)	1 (33.3%)	9 (31.0%)	**0.0009**	**0.0026**	0.5341
CHA_2_DS_2_-VASc	3.5 ± 1.5	2.9	5.1	5	3.1	< **0.0001**	> 0.9999	** < 0.0001**
Mitral regurgitation	38 (62%)	4 (16%)	14 (100%)	2 (66.7%)	18 (62%)	** < 0.0001**	**0.0017**	**0.0338**
Laboratory data								
Neutrophils (%)	64.7 ± 11	58.9	69.2	76.8	66.7	**0.0223**	**0.0171**	> 0.9999
CRP (mg/l)	6.2 ± 10.3	2.2	13.9	14.5	5.1	** < 0.0001**	0.7794	**0.0009**
proBNP (pg/ml)	1740 ± 3228	119	4356	5800	1386	** < 0.0001**	**0.0001**	**0.0353**
D-dimer (mg/l)	0.69 ± 1.01	0.32	1.19	1.91	0.63	** < 0.0001**	0.4492	**0.0026**
Creatinine (mg/dl)	1.13 ± 0.67	0.82 ± 0.21	1.38 ± 0.65	3.19 ± 1.88	0.90 ± 0.22	** < 0.0001**	0.7916	**0.0103**
Medication								
*Anticoagulation*								
IIa inhibitors	6 (8.6%)	1 (4.2%)	2 (14.3%)	2 (66.7%)	1 (3.4%)	0.1412	> 0.9999	0.2433
Xa inhibitor	32 (45.7%)	3 (12.5%)	5 (35.7%)	0	24 (82.8%)	0.2412	** < 0.0001**	**0.0005**
Vitamin K antagonist	4 (5.7%)	0	2 (14.3%)	1 (33.3%)	1 (3.4%)	0.0638	> 0.9999	0.2847
*Anti-platelet*								
Aspirin	21 (30%)	15 (62.5%)	4 (28.6%)	1 (33.3%)	1 (3.4%)	0.0578	** < 0.0001**	**0.0205**
P2Y12 inhibitors	6 (8.6%)	1 (4.2%)	1 (7.1%)	2 (66.7%)	2 (6.9%)	0.2896	> 0.9999	0.3429
DAPT	1 (1.4%)	1 (4.2%)	0	0	0	> 0.9999	0.4528	> 0.9999

### Elevated NET biomarker levels in the left atrium

We hypothesized that local blood sampling would provide a more accurate picture of the thromboinflammatory state at the left atrium due to potentially disturbed blood flow associated with enlarged left atria. We therefore compared blood sampled locally from the left atrium to peripheral blood samples collected prior to the catheterization procedure from the same patients, analyzing them for both routine clinical laboratory parameters, classical markers of inflammation and thrombosis (CRP, IL-6, D-dimers, fibrinogen), as well as specialized analysis of markers of thromboinflammation (NET biomarkers, PAD4, soluble P-selectin, VWF, ADAMTS13) and cardiac damage or fibrosis (BNP, C- terminal telopeptide of type I collagen (ICTP)).

**Fig. 2:**
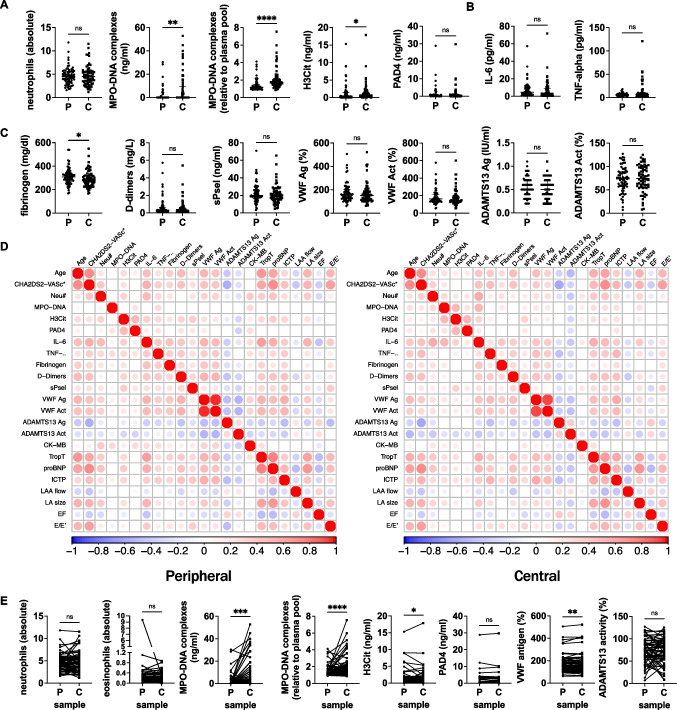
Comparison of measurements performed in peripheral venous blood or locally collected left atrial blood. **A** Neutrophil and NET parameters. **B** Pro-inflammatory cytokines. **C** Pro-thrombotic factors and VWF and ADAMTS13 measurements. **D** Spearman correlation matrices for measurements performed in peripheral blood samples (left) or central LA blood samples (right). **E** NET-related measurements in paired comparisons between peripheral and central blood samples. MPO-DNA, myeloperoxidase-DNA complexes; H3Cit, citrullinated histone H3; PAD4, peptidylarginine deiminase 4; IL-6, interleukin-6; TNF-⍺, tumor necrosis factor- ⍺; sPsel, soluble P-selectin; VWF, von Willebrand factor; ADAMTS13, a disintegrin and metalloproteinase with a thrombospondin type 1 motif, member 13; Ag, antigen; Act, activity; LA, left atrium. Mann–Whitney *U*-tests were used to determine significance in A–C and Wilcoxon tests for data in E. *P < 0.05, **P < 0.01, ****P < 0.0001; ns, not significant. *N* = 70

Neutrophil counts did not differ depending on the sampling site. Myeloperoxidase (MPO)-DNA complexes and citrullinated histone H3 (H3Cit) levels were significantly elevated in samples drawn from the left atrium compared to peripheral samples (Fig. 2A). Classical pro-inflammatory cytokines IL-6 and TNFα were similar in both sampling types (Fig. 2B). Although there was a modest reduction in fibrinogen in the central sample, the remainder of factors associated with a prothrombotic phenotype or endothelial activation were similar between the two types of samples (Fig. 2C). We generated correlation matrices to identify patterns in correlations in blood samples drawn through venipuncture or during catheterization directly from the left atrium (Fig. 2D). The profile is visibly similar in samples collected peripherally versus within the heart. We also performed paired analyses on the thromboinflammation parameters and confirmed similar findings as in Fig. 2A–C (Fig. 2E). As the levels of NETs were higher in the blood sample collected in the left atrium, we proceeded with all remaining analyses with these samples only.

### Distinct thromboinflammatory profiles based on procedure type

We next analyzed the same parameters according to the type of procedure that was performed, combining MitraClip and LAAC patients into a combined cohort based on similar clinical characteristics (high presence of atrial fibrillation, NTproBNP, CRP, and CHA_2_DS_2_-VASc score). Here, neutrophil counts were highest in the MC + LAAC cohort as compared to PFO or PVA but there was no difference in levels of NETs with the exception of higher H3Cit in MC + LAAC compared to PFO patients (Figure S1A). MC + LAAC and PVA had higher IL-6 compared to PFO, whereas TNF-alpha was elevated only in MC + LAAC compared to PFO patients (Figure S1B). Notably, fibrinogen was the only pro-thrombotic marker that was not elevated in MC + LAAC patients (Figure S1C). Von Willebrand factor activity and antigen were highly elevated in MC + LAAC patients with a mean of twice normal-range values. Accordingly, ADAMTS13 activity and antigen levels were reduced in MC + LAAC but not in PVA patients, compared to the PFO cohort.

Analysis of troponin T, proBNP, and ICTP confirmed a higher degree of cardiac damage or fibrotic remodeling in MC + LAAC patients, with PVA patients having only elevated troponin T or proBNP compared to PFO patients (Figure S1D). Furthermore, echocardiographic measurements of body surface area corrected- left atrial size (left atrial volume index, LAVI) or LA flow velocity, as well as ejection fraction and E/E’ ratio were analyzed for each patient. MC + LAAC patients had reduced LA flow velocity, enlarged left atria, higher E/E’ compared to both PVA and PFO cohorts, and lower EF compared only to the PFO cohort (Figure S1E).

Spearman correlation coefficients were calculated for the different parameters measured above, with correlation matrices shown in Figure S2. In contrast to the sampling type, there was a clear difference in the correlation matrix depending on the procedure type. Heat maps showed distinct profiles among the patient groups with more significantly positive correlations between thrombotic markers, cardiac damage biomarkers, and NET measurements were seen in the MC + LAAC group (Figure S1F). Finally, a principal component analysis was performed with all study measurements described above to show the clustering the individual patients according to procedure type (Figure S1G). This analysis showed that the PFO group clustered distinctly from the others, with less variation in parameters. The greatest degree of variation in patient profile distribution was evident in the MC + LAAC cohort, with PVA patients clustering between the two. The biplot which shows the contribution of each measured parameter to the PCA is shown in Figure S3. In summary, we could identify differences in the measured blood parameters depending on the procedure type, along with expected differences in cardiac parameters using biomarkers in blood or imaging.

### Chronicity of atrial fibrillation correlates with thromboinflammatory profile

**Fig. 3:**
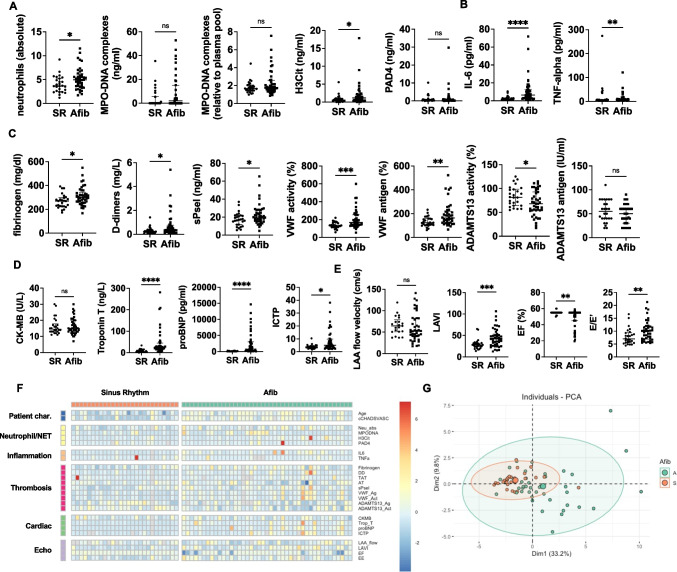
Comparison of left atrial blood measurements and cardiac parameters in patients with sinus rhythm (SR) or atrial fibrillation (AF). **A** Neutrophil and NET parameters. **B** Pro-inflammatory cytokines. **C** Pro-thrombotic factors and VWF and ADAMTS13 measurements. **D** Biomarkers of cardiac damage/remodeling. **E** Structural and functional measurements as performed by echocardiography. **F** Heat map showing reduced dimensionality data with relative Z-scores for each parameter. **G** Principal component analysis (PCA) showing clustering of procedure groups by variance. MPO-DNA, myeloperoxidase-DNA complexes; H3Cit, citrullinated histone H3; PAD4, peptidylarginine deiminase 4; IL-6, interleukin-6; TNF-⍺, tumor necrosis factor- ⍺; sPsel, soluble P-selectin; VWF, von Willebrand factor; ADAMTS13, a disintegrin and metalloproteinase with a thrombospondin type 1 motif, member 13; Ag, antigen; Act, activity; LA, left atrium; CK-MB, creatine kinase MB; proBNP, brain natriuretic peptide; ICTP, C-telopeptide of collagen I; LAA, left atrial appendage; LAVI, left atrial volume index; EF, ejection fraction. SR, *N* = 27; AF, *n* = 43. Mann–Whitney *U*-tests were used to determine significance. *P < 0.05, **P < 0.01, ***P < 0.001; ****P < 0.0001; ns, not significant

Finally, we performed similar analyses by grouping patients according to the presence or absence of atrial fibrillation (AF). Similar results were obtained to the LAVI cutoff stratification, with an additional significant increase in H3Cit, IL-6, D-dimers, soluble P-selectin, and ICTP in patients with any history of atrial fibrillation (Fig. 3A–D). The ejection fraction was also significantly reduced in the AF group (Fig. 3E). The heterogeneity across the AF patients and the PCA plots was similar to what was seen in patients with enlarged LAVI (Fig. 3F, G). Correlation matrices for all parameters measured for patients in SR and any AF are depicted in Figure S4. We also confirmed similar findings in the peripheral blood samples collected by traditional venipuncture (Figure S5).

In parallel, we also performed a different subgroup analysis where we classified the AF patients based on permanent AF status and including the intermittent AF patients together with those who are in sinus rhythm, as at the time of blood sampling these patients were also in sinus rhythm (SR) (Figure S6). In contrast to the classification with all AF patients, with examination of permanent AF only lost the distinct profile based on our measured parameters and several of the significant differences between the AF and SR, including VWF/ADAMTS13 measurements. This indicates that a prior history of AF episodes rather than active AF at the time of sampling is more predictive of thromboinflammation.

### Correlations between NETs, VWF/ADAMTS13 *axis*, and cardiac injury with LA size

**Fig. 4:**
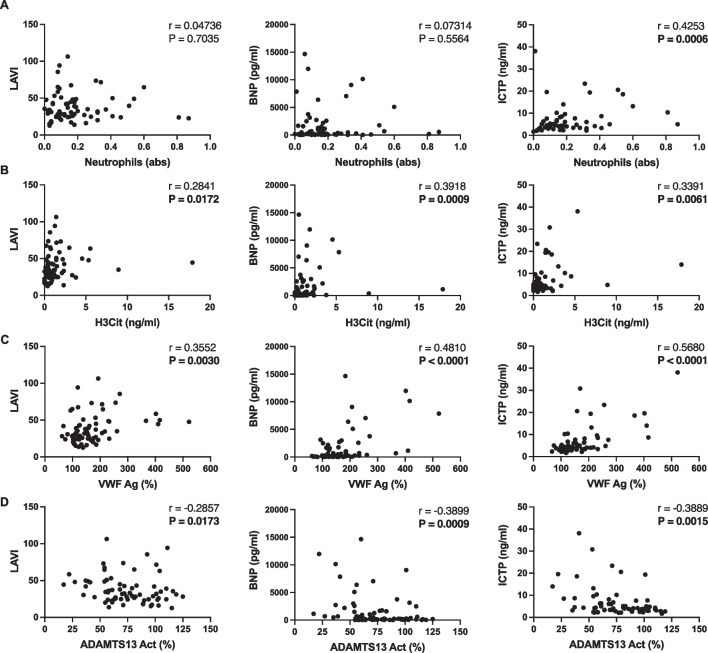
Spearman correlation analyses of left ventricular volume index (LAVI) and cardiac injury biomarkers with selected thromboinflammation parameters. Neutrophil counts (**A**), citrullinated histones (**B**), and VWF antigen (**C**) positively correlated with LAVI, proBNP, and ICTP. Conversely, ADAMTS13 activity (**D**) negatively correlated with LAVI, proBNP, and ICTP. H3Cit, citrullinated histone H3; VWF, von Willebrand factor; ADAMTS13, a disintegrin and metalloproteinase with a thrombospondin type 1 motif, member 13; Ag, antigen; Act, activity; *r*, Spearman correlation coefficient. *N* = 70

Individual Spearman correlation plots are shown in Fig. 4. Neutrophil numbers and H3Cit levels correlated positively with LAVI, proBNP, and ICTP (Fig. 4A, B), indicating a link with fibrosis development and ongoing cardiac damage. VWF antigen was also significantly positively correlated with the same factors, with ADAMTS13 activity conversely negatively correlated (Fig. 4C, D). PAD4 levels correlated with reduced ADAMTS13 activity levels correlated with higher values throughout all NET markers in LA blood (Fig. 1D), and PAD4, which has been shown to render ADAMTS13 inactive [22]. This suggests a potential role for PAD4-mediated citrullination of ADAMTS13 in its reduced activity, in addition to lower levels of circulating antigen.

### Enlarged LAVI correlates with a distinct thromboinflammatory profile

**Fig. 5:**
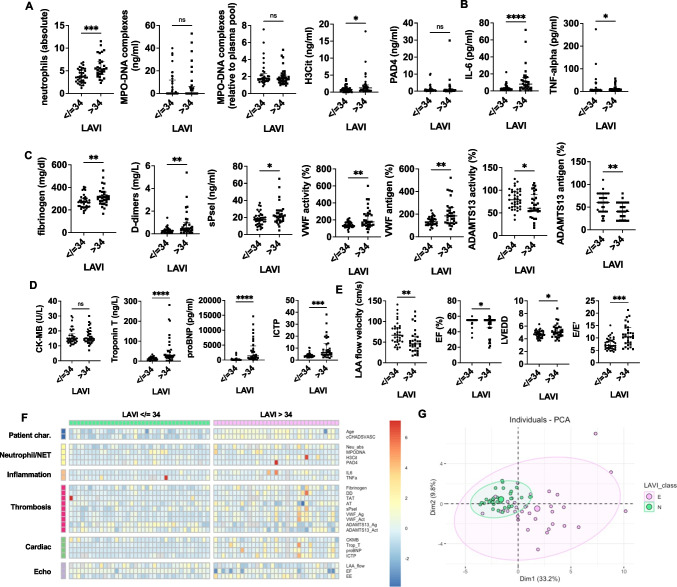
Comparison of left atrial blood measurements and cardiac parameters in patients with normal versus enlarged left atria (LA). Patients were stratified by LAVI with 34 as a cutoff for enlarged LA. **A** Neutrophil and NET parameters. **B** Pro-inflammatory cytokines. **C** Pro-thrombotic factors and VWF and ADAMTS13 measurements. **D** Biomarkers of cardiac damage/remodeling. **E** Structural and functional measurements as performed by echocardiography. **F** Heat map showing reduced dimensionality data with relative Z-scores for each parameter. **G** Principal component analysis showing clustering of procedure groups by variance. MPO-DNA, myeloperoxidase-DNA complexes; H3Cit, citrullinated histone H3; PAD4, peptidylarginine deiminase 4; IL-6, interleukin-6; TNF-⍺, tumor necrosis factor- ⍺; sPsel, soluble P-selectin; VWF, von Willebrand factor; ADAMTS13, a disintegrin and metalloproteinase with a thrombospondin type 1 motif, member 13; Ag, antigen; Act, activity; LA, left atrium; CK-MB, creatine kinase MB; proBNP, brain natriuretic peptide; ICTP, C-telopeptide of collagen I; LAA, left atrial appendage; LAVI, left atrial volume index; EF, ejection fraction. Normal atria (N), *n* = 37; enlarged atria (E), *n* = 33. Mann–Whitney *U*-tests were used to determine significance. *P < 0.05, **P < 0.01, ***P < 0.001; ****P < 0.0001; ns, not significant. *N* = 70

In parallel, echocardiographic measurements including left atrium size and flow velocity, as well as ejection fraction of the left ventricle and E/E’ were recorded and analyzed. A cutoff for enlarged left atria using a left atrial volume index of greater than 34 was used according to European Society on Cardiology guidelines [10]. Here, neutrophil numbers, H3Cit, TNFα, and IL-6, but not MPO-DNA, were elevated in the enlarged LAVI subgroup (Fig. 5A, 5B). In the thrombotic factor measurements, once again VWF activity and antigen were both highly elevated, with a concurrent reduction in ADAMTS13 activity and antigen (Fig. 5C). The cardiac damage markers were higher in those patients with large LAVI, and LA flow velocity was reduced while E/E’ was increased in the patient cohort (Fig. 5D, E). We also performed correlation analyses with the measured biochemical parameters in blood and found that there was heterogeneity in the profiles across the different donors (Fig. 5F). The PCA also identified differences in the profiles of the normal vs larger LA samples (Fig. 5G). As the PFO cohort had significantly smaller LAVI, we also performed a subgroup analysis without the inclusion of these patients. Here, we also saw a similar increase in neutrophils, IL-6, VWF, and a subsequent decrease in ADAMTS13 parameters (Figure S7).

In univariable linear regression analyses, age (β, 0.39; CI, 0.37–1.14, *p* < 0.001), neutrophils (β, 0.39; CI, 0.17–0.64, *p* = 0.001), VWF Ag (β, 0.34; CI, 0.12–0.64, *p* = 0.004), and H3Cit (β, 0.31; CI, 0.03–0.20, *p* = 0.01) were associated with LAVI. Added to a multivariable model with LAVI as dependent variable, age (β; 0.26; CI, 0.004–0.95, *p* = 0.048) and neutrophil count (β; 0.32; CI, 0.06–0.63, *p* = 0.017) remained associated with LAVI, but VWF Ag (β; 0.065; CI, − 0.24–0.38, *p* = 0.65) and H3Cit (β; 0.068; CI, − 0.07–0.12, *p* = 0.59) were no longer associated with LAVI. In a subgroup analysis with the same multivariate analysis without the PFO cohort, only neutrophil count remained significant (Table S1). In a multiple regression analysis with NETs as the dependent variables, neutrophil counts (*p* = 0.0493), PAD4 (*p* = 0.0066), D-dimer (*p* = 0.0142), VWF antigen (*p* < 0.001), proBNP (*p* = 0.0322), and E/E’ (*p* = 0.0491) independently associated with H3Cit whereas they did not associate with MPO-DNA.

Finally, we followed up with patients over the 12 months following initial inclusion in the study to identify thrombotic events or major bleeding episodes. Two patients died in the year after their procedure. One patient suffered from a femoral fracture and acute kidney failure, whereas the second had progressive dysphagia of multifactorial origin including an acute ischemic stroke. Two patients suffered an acute ischemic stroke, and eight patients suffered from bleeding events. The low number of events precluded further analysis within the study design.

## Discussion

In this prospective study of patients undergoing elective invasive cardiac interventions, we measured blood markers of thromboinflammation peripherally and in the left atrium and correlated these data with sonographic measures of anatomical and functional abnormalities of the left atrium, a structure implicated in ischemic stroke of undetermined etiology. We identified that blood samples collected locally within the left atrium had higher levels of NETs, but that other measured blood parameters did not differ from peripheral blood samples. It has been hypothesized that central blood sampling may provide more information compared to standard venipuncture. As this is invasive and only possible during catheterization procedures, being able to rely on peripheral blood samples (with the likely exception of NET measurements) allows for greater future applicability of this concept in clinical care.

Blood collected from the LA required removal of blood through a catheter, and therefore blood sampling was procedurally different as compared to venipuncture. It is unlikely that shear stress from blood traveling thorugh the catheter led to elevated NET levels, as the inner diameter of the catheter is smaller in the PFO/ASD procedure as compared to PVA/MC/LAAC and NET levels were higher in the latter. This study was performed within the context of clinically indicated material, but withdrawal through similar methodology and similarly sized tubing would have been preferable.

We have performed NET measurements according to recent recommendations from the International Society on Thrombosis and Haemostasis reported at the ISTH2022 Congress Vascular Biology SSC session [13] including measurement of MPO-DNA complexes and citrullinated histones. Of note, these are exploratory research methods that are not validated for clinical use and have limitations in detection thresholds. We cannot rule out the contribution of eosinophil extracellular traps, which also could provide a signal for H3Cit [14], in our study as there is currently no established method to assess EETs with adequate sensitivity in plasma. However, neutrophil counts significantly correlated with H3Cit levels, whereas eosinophil counts did not (Figure S8). We did not identify strong correlations with previously established secreted platelet factors and NET release in our study, and the heparin treatment accompanying catheterization procedures precluded accurate PF4 measurements. Expanding to include additional cytokines and circulating factors associated with more NET release (including HMGB1, IL-17) may be of value in future studies. Assessing the ability of plasma from patients to induce NET formation ex vivo in healthy volunteer neutrophils may provide supporting information about a systemic environment that could be promoting NET release, even if NET fragments themselves are low or not detectable with ELISA-based methods [9]. This may be further elevated in plasma prepared from blood samples collected within the heart, as our study showed increased NETs in LA blood compared to peripheral blood.

### Inclusion of thromboinflammation parameters in clinical care?

VWF/ADAMTS13 measurements are already available in most clinical hematology labs. The measurements used in this study for NETs are not validated for clinical or diagnostic use. Recently, a new assay to measure nucleosomes in human plasma has been CE-marked (Nu.Q NETs assay). There are currently no clinical diagnostic recommendations to measure NETs in any human disease conditions, likely due to lack of an available clinical-grade test. There is still work to be done to evaluate where and when NET measurements may be of either prognostic or diagnostic value in patients. Based on preclinical results, in many cases, elevated NETs are likely to be found in a broad variety of disease states, and may be either causative or a reflection of ongoing other etiologies.

Correlations show that there may be some prognostic value in generating a thromboinflammatory profile in the context of cardiac parameters, including clinical laboratory measurements such as NTproBNP, ICTP, and troponin T. There is also an established algorithm to interpret different components of a thromboinflammatory profile and how these may differ in different diseases. The implementation of artificial intelligence-based or machine learning approaches may be able to tackle this question in the near future, but would first require routine implementation of the individual laboratory testing in a broader capacity.

### Utility of LA size measurements

There may also be added value in performing echocardiographic measurements to assess structural and functional factors in the left atrium. Secondary analysis of the STROKE-AF trial showed that left atrial enlargement was one of the few predictors of AF detection upon multivariate analysis [20]. Our patient cohort with LAVI > 34 had a 90.9% incidence of AF overall, with 39.4% having permanent AF. This study supports that LA size may be of value to measure in patients with an elevated thromboinflammatory profile, or vice versa. This non-invasive measurement uses transthoracic rather than transesophageal echo and may be preferable to less sensitive measurements including monitoring for AF. The ARCADIA trial with randomization of 100 patients on apixaban or aspirin assessed if one is superior in preventing stroke recurrence in patients with atrial cardiopathy, and found no superiority of apixaban [7]. There was also no difference in bleeding episodes. This trial thus provides valuable information about anticoagulation in patients with enlarged left atria, and further highlights the need for novel therapeutic strategies in these patients, as anticoagulation or platelet targeting may not be sufficient in the case of NET-mediated thrombosis.

### Independence from current AF status

In this study, we collected data about atrial fibrillation status at the time of procedure, as well as long-term history that each patient had had with potential prior episodes of AF. Any prior history of atrial fibrillation as good if not the best indication of an elevated thromboinflammatory profile. When distinguishing patients with only permanent AF, then the distinction between the two patient populations is lost, and the thromboinflammatory profile remains similar in either patient group. This could be due to medication or intervention that is successfully treating the AF, or early or less severe arrhythmias having a reduced thromboinflammatory profile that is balanced out when accounting for the difference in disease severity.

### Limitations

Our study has limitations. First, patient recruitment was slowed as it coincided with intermittent periods of COVID-19 lockdown in Germany. Secondly, the study was originally designed to follow up patients for a 2-year period with repeat blood sampling. Due to the timing of the COVID-19 pandemic waves, this sampling was not performed due to insufficient ability to recall patients to the hospital for elective blood sampling. The limited sample size did not allow for correlation with outcome events (e.g., stroke). The study was therefore not sufficiently powered for secondary analysis of bleeding or thrombotic events with respect to the parameters analyzed at the time of study inclusion. The study was also not sufficiently powered to identify the impact of differences in medication, including antiplatelet therapy and anticoagulation, or to make conclusions about the lack of AF in patients with enlarged LA. Similarly, the analysis could not be extensively adjusted for factors potentially associated with increased LAVI.

LA strain, which would have been a valuable addition to the study, was not measured as it was not in regular clinical use at the time of the study’s echocardiographic measurements. Thrombosis incidence noted in the 1-year follow-up could not be ruled out as preexisting as imaging was not conducted at the time of study enrollment, and de novo asymptomatic thrombosis may have been missed. Furthermore, although patients with PFO closure were assessed for AF by 3-day Holter EKG monitoring, and all PVA patients had previously been diagnosed with AF, no systematic atrial fibrillation screening was performed for patients undergoing LAAC or MC placement. To do this, it would have required either Holter monitoring or a subcutaneously implanted reveal device, which was not clinically indicated. Therefore, atrial remodeling due to known inflammatory processes or due to atrial arrhythmopathies without documented atrial fibrillation cannot be ruled out.

The control cohort in this study is not age-matched and had a higher incidence of aspirin use due to prior stroke associated with their PFO. Older patients are generally not eligible for PFO closure.

PVA patients also received more Xa inhibitors than either the PFO or the MC and LAAC cohorts. This study was not designed to analyze the impact of medication use, but this would be of interest to pursue in future studies with larger patient numbers.

Finally, comparisons were made between left atrial blood and peripheral venous blood, and not right atrial or arterial blood draws. This was done to avoid introducing additional risk to the patient, as a right atrial draw would have involved additional procedure time, and left atrial blood was already being withdrawn as part of the procedure (generally discarded, in our case collected). The translatability of thromboinflammatory profiling to future clinical practice was a reason for opting for traditional venipuncture to collect the systemic blood samples. We also did not include a healthy volunteer cohort due to lack of access to LA blood as this would not be clinically indicated.

## Conclusion

Blood drawn from the left atrium from patients undergoing elective heart interventions shows significantly higher levels of specific (not all) thrombotic markers as compared to blood drawn from peripheral veins, and a relationship between NETs and dysregulation of the VWF-ADAMTS13 axis. In those with evidence of enlarged left atria on echocardiography, left atrial NETS and VWF were significantly increased compared to those within normal LAVI ranges, indicative of a potential role of NETs and VWF in a local left atrial thromboembolic environment. This may help identify patients at risk for cardioembolic events who could be treated with preventive therapy based on left atrial measurements.

## Supplementary Information

Below is the link to the electronic supplementary material.

Supplementary file1 Table S1. Multivariate analysis of LAVI with age, neutrophils, VWF antigen, and H3Cit, excluding patients with PFO in the analysis. *N*=42. Figure S1. Comparison of left atrial blood measurements and cardiac parameters grouped by type of procedure performed. Patients underwent either patent foramen ovale closure (PFO), MitraClip placement or left atrial appendage closure (LAAC), or pulmonary vein ablation (PVA). A. Neutrophil and NET parameters. B. Pro-inflammatory cytokines. C. Pro-thrombotic factors and VWF and ADAMTS13 measurements. D. Biomarkers of cardiac damage/remodeling. E. Structural and functional measurements as performed by echocardiography. F. Heat map showing reduced dimensionality data with relative Z-scores for each parameter. G. Principal component analysis showing clustering of procedure groups by variance. MPO-DNA, myeloperoxidase-DNA complexes; H3Cit, citrullinated histone H3; PAD4, peptidylarginine deiminase 4; IL-6, interleukin-6; TNF-⍺, tumor necrosis factor- ⍺; sPsel, soluble P-selectin; VWF, von Willebrand factor; ADAMTS13, a disintegrin and metalloproteinase with a thrombospondin type 1 motif, member 13; Ag, antigen; Act, activity; LA, left atrium; CK-MB, creatine kinase MB; proBNP, brain natriuretic peptide; ICTP, C-telopeptide of collagen I; LAA, left atrial appendage; LAVI, left atrial volume index; EF, ejection fraction. PFO *n* = 24, MC+LAAC *n* = 17, PVA *n* = 29. Kruskal-Wallis with Dunn’s post tests were used to determine significance. *P < 0.05, **P < 0.01, ***P<0.001; ****P<0.0001, ns = not significant. Figure S2. Spearman correlation analyses of all parameters measured in the study stratified by catheterization procedure. Figure S3. Principal component analysis (PCA) plots (A,C) and their respective biplots (B,D) for stratification by procedure. Panels A and B show the analysis with all parameters of the study included. Panels C and D show analysis performed only for thromboinflammation and cardiac injury parameters. Figure S4. Spearman correlation analyses of all parameters measured in the study stratified by presence of atrial fibrillation (AF) or absence of atrial fibrillation (sinus rhythm, SR). Figure S5. Comparison of peripheral blood measurements and cardiac parameters in patients with sinus rhythm (SR) or atrial fibrillation (AF). A. Neutrophil and NET parameters. B. Pro-inflammatory cytokines. C. Pro-thrombotic factors and VWF and ADAMTS13 measurements. D. Biomarkers of cardiac damage/remodeling. E. Heat map showing reduced dimensionality data with relative Z-scores for each parameter. F. Principal component analysis showing clustering of procedure groups by variance. MPO-DNA, myeloperoxidase-DNA complexes; H3Cit, citrullinated histone H3; PAD4, peptidylarginine deiminase 4; IL-6, interleukin-6; TNF-⍺, tumor necrosis factor- ⍺; sPsel, soluble P-selectin; VWF, von Willebrand factor; ADAMTS13, a disintegrin and metalloproteinase with a thrombospondin type 1 motif, member 13; Ag, antigen; Act, activity; LA, left atrium; CK-MB, creatine kinase MB; proBNP, brain natriuretic peptide; ICTP, C-telopeptide of collagen I; LAA, left atrial appendage; LAVI, left atrial volume index; EF, ejection fraction. SR, *N* = 27; AF, *n* = 43. Mann-Whitney U-tests were used to determine significance. *P < 0.05, **P < 0.01, ***P<0.001; ****P<0.0001, ns = not significant. Figure S6. Comparison of patients with permanent atrial fibrillation compared to patients with sinus rhythm at the time of blood sampling (SR). A. Neutrophil and NET parameters. B. Pro-inflammatory cytokines. C. Pro-thrombotic factors and VWF and ADAMTS13 measurements. D. Biomarkers of cardiac damage/remodeling. E. Structural and functional measurements as performed by echocardiography. F. Heat map showing reduced dimensionality data with relative Z-scores for each parameter. G. Principal component analysis showing clustering of procedure groups by variance. MPO-DNA, myeloperoxidase-DNA complexes; H3Cit, citrullinated histone H3; PAD4, peptidylarginine deiminase 4; IL-6, interleukin-6; TNF-⍺, tumor necrosis factor- ⍺; sPsel, soluble P-selectin; VWF, von Willebrand factor; ADAMTS13, a disintegrin and metalloproteinase with a thrombospondin type 1 motif, member 13; Ag, antigen; Act, activity; LA, left atrium; CK-MB, creatine kinase MB; proBNP, brain natriuretic peptide; ICTP, C-telopeptide of collagen I; LAA, left atrial appendage; LAVI, left atrial volume index; EF, ejection fraction. SR, *N* = 57; Permanent AF, *N* = 13. Mann-Whitney U-tests were used to determine significance. *P < 0.05, **P < 0.01, ***P<0.001; ****P<0.0001, ns = not significant. Figure S7. Comparison of left atrial blood measurements and cardiac parameters in patients with normal versus enlarged left atria. A. Neutrophil and NET parameters. B. Pro-inflammatory cytokines. C. Pro-thrombotic factors and VWF and ADAMTS13 measurements. D. Biomarkers of cardiac damage/remodeling. E. Structural and functional measurements as performed by echocardiography. F. Heat map showing reduced dimensionality data with relative Z-scores for each parameter. G. Principal component analysis showing clustering of procedure groups by variance. MPO-DNA, myeloperoxidase-DNA complexes; H3Cit, citrullinated histone H3; PAD4, peptidylarginine deiminase 4; IL-6, interleukin-6; TNF-⍺, tumor necrosis factor- ⍺; sPsel, soluble P-selectin; VWF, von Willebrand factor; ADAMTS13, a disintegrin and metalloproteinase with a thrombospondin type 1 motif, member 13; Ag, antigen; Act, activity; LA, left atrium; CK-MB, creatine kinase MB; proBNP, brain natriuretic peptide; ICTP, C-telopeptide of collagen I; LAA, left atrial appendage; LAVI, left atrial volume index; EF, ejection fraction. Normal atria, *n* = 16; enlarged atria, *n* = 30. Mann-Whitney U-tests were used to determine significance. *P < 0.05, **P < 0.01, ****P<0.0001, ns = not significant. Figure S8. Spearman correlation analyses of left ventricular volume index (LAVI) and cardiac injury biomarkers with eosinophils. Neutrophil, but not eosinophil counts were significantly correlated with citrullinated histones (A). Eosinophil counts were positively correlated with ICTP but not with LAVI or proBNP. H3Cit, citrullinated histone H3; *r* = Spearman correlation coefficient. *N* = 65-67. (PDF 2241 KB)

## Data Availability

The full data from this study comprises personal data and is available with a data sharing agreement to be requested from the corresponding author.
