# Supplementary material for: NET burden in left atrial blood is associated with biomarkers of thrombosis and cardiac injury in patients with enlarged left atria
Source: Clin Res Cardiol. 2024 Jun 26;114(1):112–25. doi: 10.1007/s00392-024-02464-9 (PMC11772398; doi:10.1007/s00392-024-02464-9)
Supplement: Supplementary file 1 — Supplementary file1 Table S1. Multivariate analysis of LAVI with age, neutrophils, VWF antigen, and H3Cit, excluding patients with PFO in the analysis. N=42. Figure S1. Comparison of left atrial blood measurements and cardiac parameters grouped by type of procedure performed. Patients underwent either patent foramen ovale closure (PFO), MitraClip placement or left atrial appendage closure (LAAC), or pulmonary vein ablation (PVA). A. Neutrophil and NET parameters. B. Pro-inflammatory cytokines. C. Pro-thrombotic factors and VWF and ADAMTS13 measurements. D. Biomarkers of cardiac damage/remodeling. E. Structural and functional measurements as performed by echocardiography. F. Heat map showing reduced dimensionality data with relative Z-scores for each parameter. G. Principal component analysis showing clustering of procedure groups by variance. MPO-DNA, myeloperoxidase-DNA complexes; H3Cit, citrullinated histone H3; PAD4, peptidylarginine deiminase 4; IL-6, interleukin-6; TNF-⍺, tumor necrosis factor- ⍺; sPsel, soluble P-selectin; VWF, von Willebrand factor; ADAMTS13, a disintegrin and metalloproteinase with a thrombospondin type 1 motif, member 13; Ag, antigen; Act, activity; LA, left atrium; CK-MB, creatine kinase MB; proBNP, brain natriuretic peptide; ICTP, C-telopeptide of collagen I; LAA, left atrial appendage; LAVI, left atrial volume index; EF, ejection fraction. PFO n = 24, MC+LAAC n = 17, PVA n = 29. Kruskal-Wallis with Dunn’s post tests were used to determine significance. *P < 0.05, **P < 0.01, ***P<0.001; ****P<0.0001, ns = not significant. Figure S2. Spearman correlation analyses of all parameters measured in the study stratified by catheterization procedure. Figure S3. Principal component analysis (PCA) plots (A,C) and their respective biplots (B,D) for stratification by procedure. Panels A and B show the analysis with all parameters of the study included. Panels C and D show analysis performed only for thromboinflammation and cardiac injury parameters. [file 392_2024_2464_MOESM1_ESM.pdf]

Figure S1

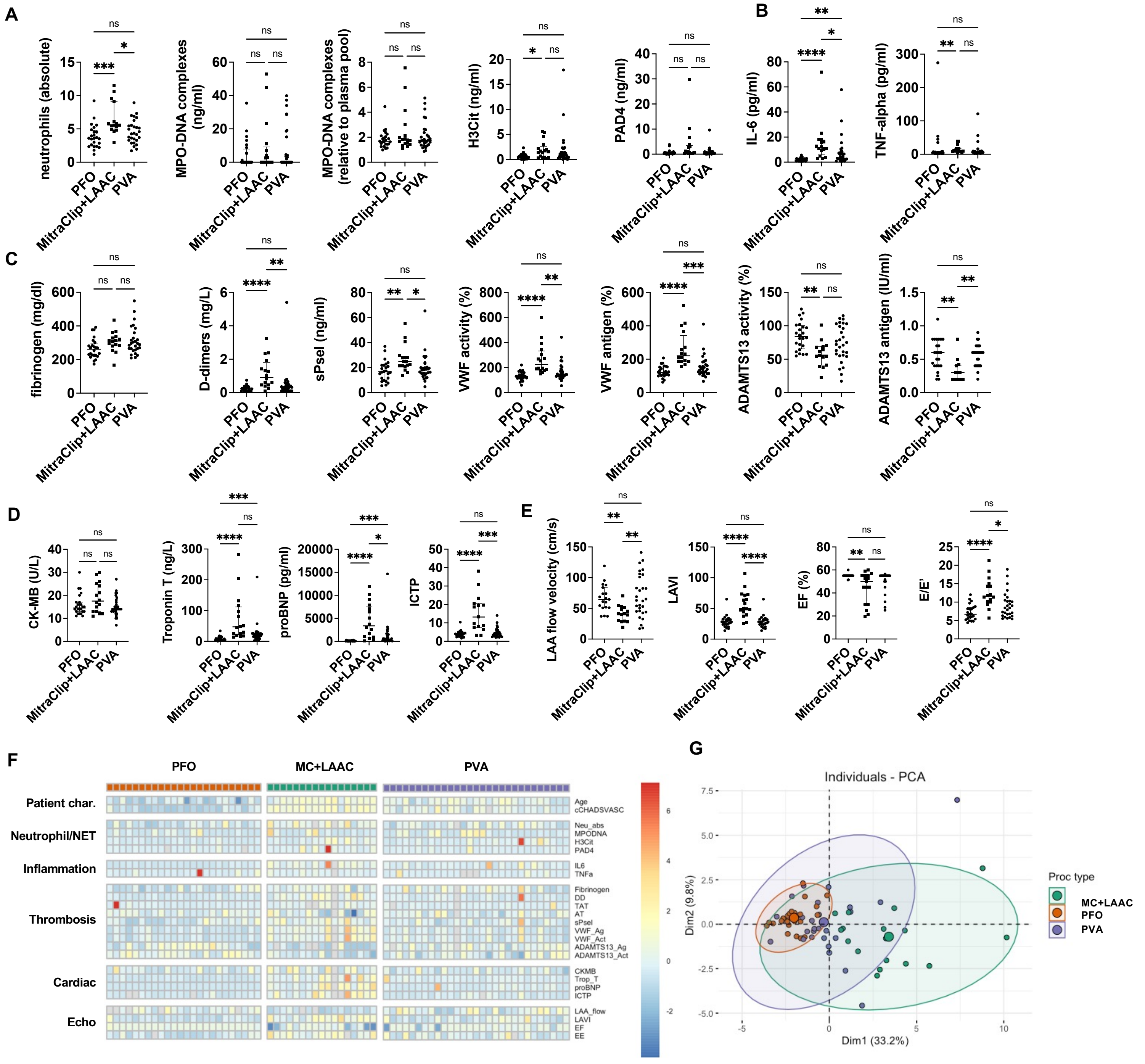

A
B
C
D
E
F
G
neutrophils (absolute)
MPO-DNA complexes (ng/ml)
MPO-DNA complexes (relative to plasma pool)
H3Cit (ng/ml)
PAD4 (ng/ml)
IL-6 (pg/ml)
TNF-alpha (pg/ml)
fibrinogen (mg/dl)
D-dimers (mg/L)
sPsel (ng/ml)
VWF activity (%)
VWF antigen (%)
ADAMTS13 activity (%)
ADAMTS13 antigen (IU/ml)
CK-MB (U/L)
Troponin T (ng/L)
proBNP (pg/ml)
ICTP
LAA flow velocity (cm/s)
LAVI
EF (%)
E/E'
PFO
MitraClip+LAAC
PVA
ns
PFO
MC+LAAC
PVA
Patient char.
Neutrophil/NET
Inflammation
Thrombosis
Cardiac
Echo
Age
cCHADSVASC
Neu_abs
MPODNA
H3Cit
PAD4
IL6
TNFa
Fibrinogen
DD
TAT
AT
sPsel
VWF_Ag
VWF_Act
ADAMTS13_Ag
ADAMTS13_Act
CKMB
Trop_T
proBNP
ICTP
LAA_flow
LAVI
EF
EE
Individuals - PCA
Dim1 (33.2%)
Dim2 (9.8%)
Proc type
MC+LAAC
PFO
PVA

Figure S2

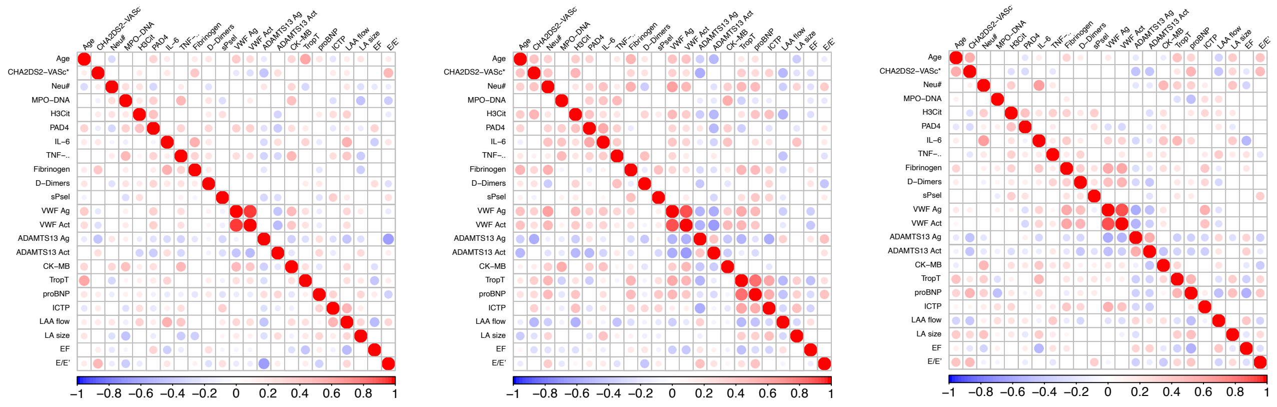

PFO closure | **MitraClip + LAAC** | **Pulmonary vein ablation**

Figure S3

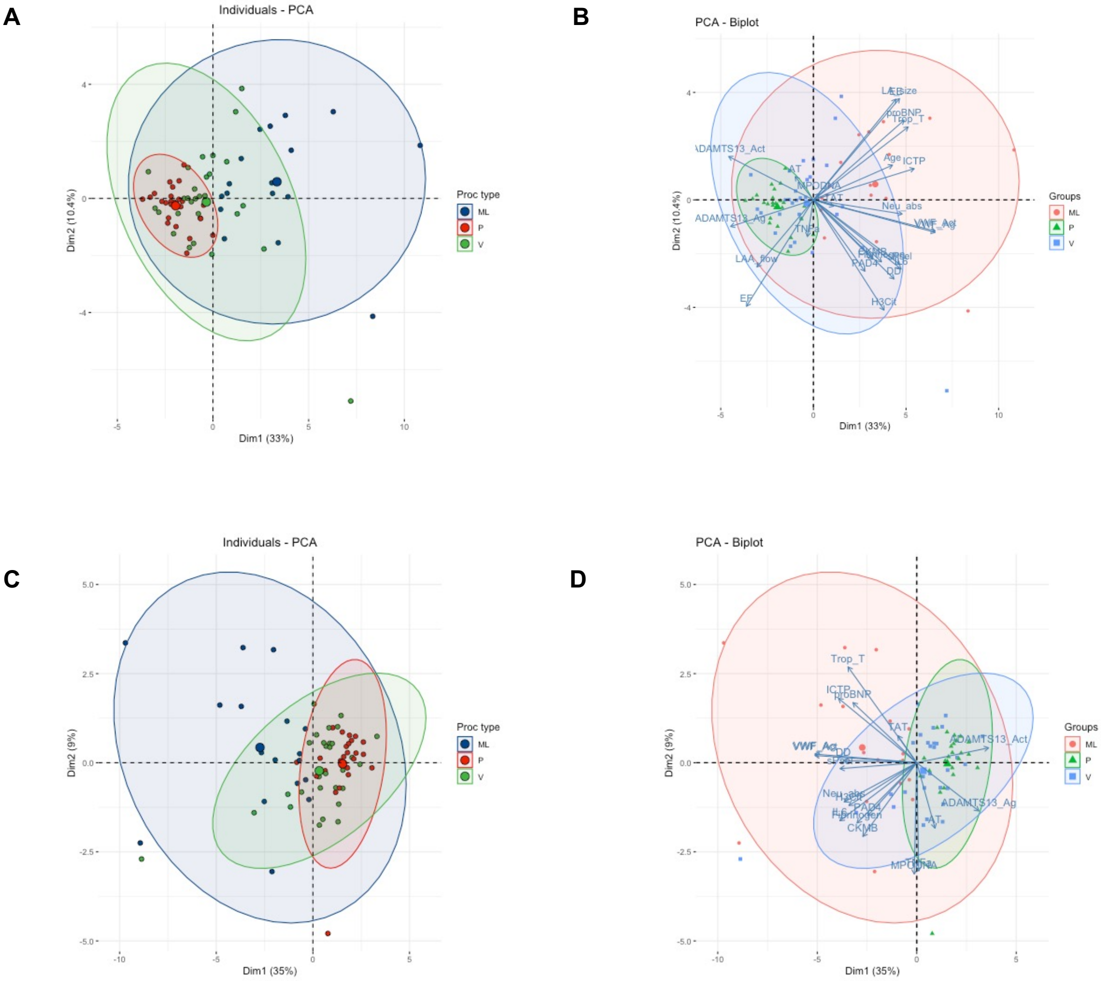

Figure S4

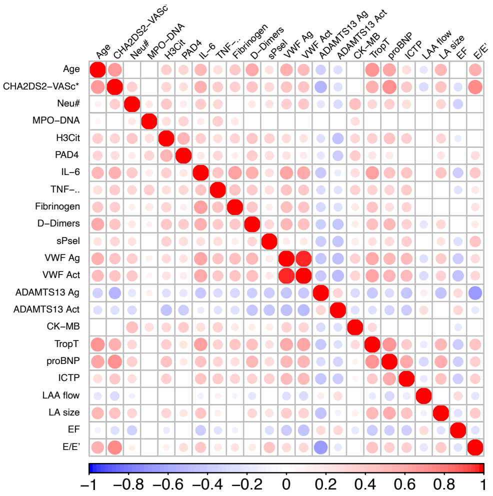

Sinus rhythym

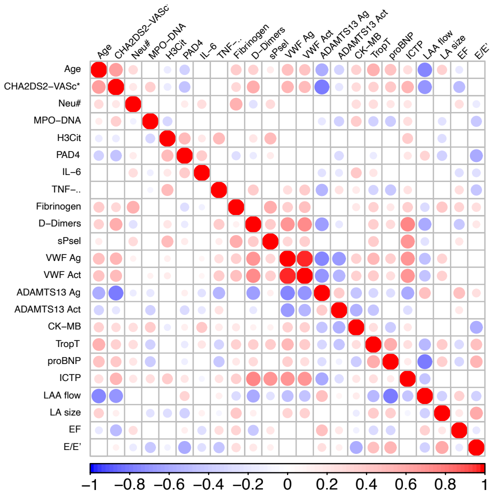

Afib

Figure S5

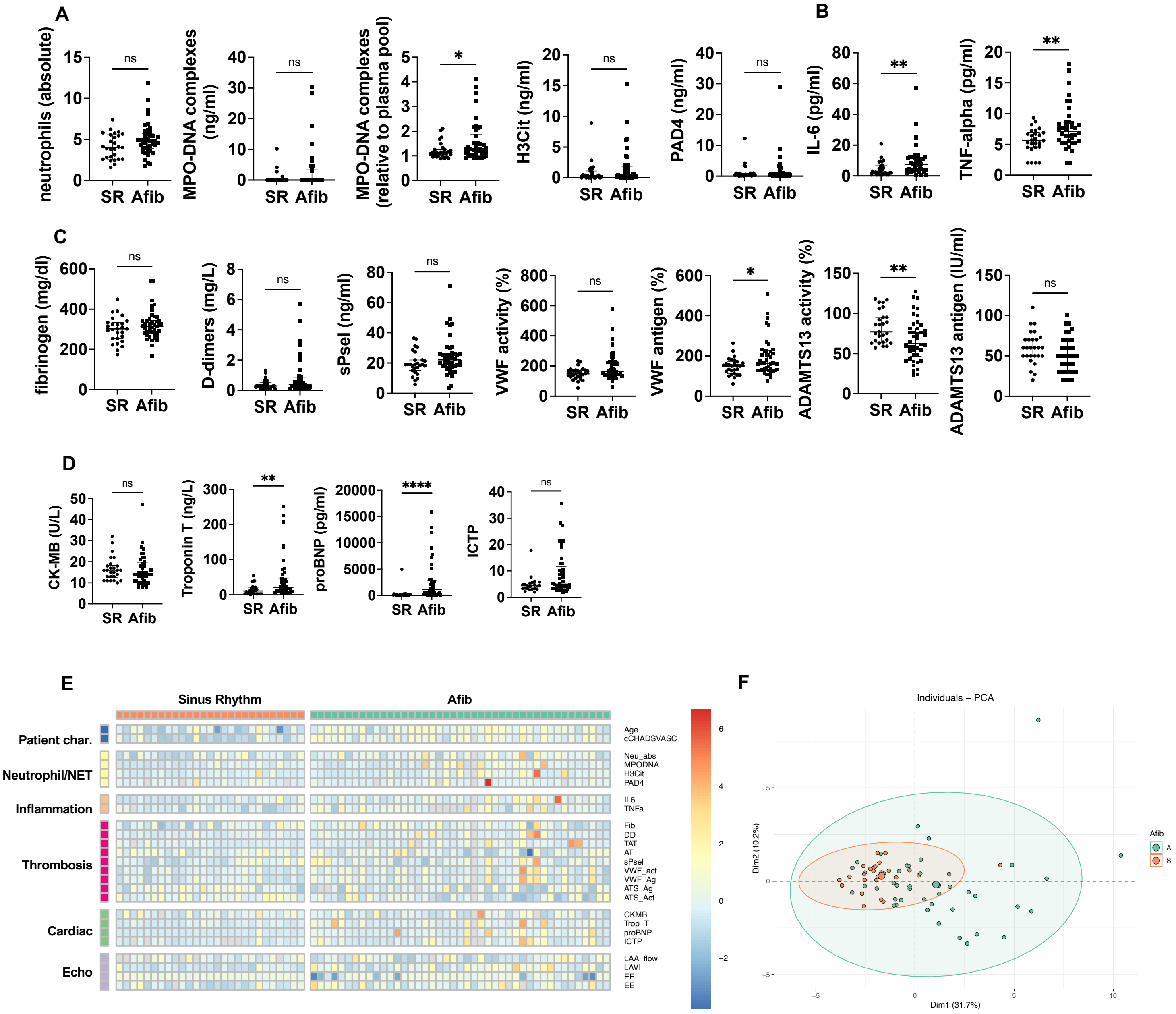

A
B
C
D
E
F
neutrophils (absolute)
MPO-DNA complexes (ng/ml)
MPO-DNA complexes (relative to plasma pool)
H3Cit (ng/ml)
PAD4 (ng/ml)
IL-6 (pg/ml)
TNF-alpha (pg/ml)
fibrinogen (mg/dl)
D-dimers (mg/L)
sPsel (ng/ml)
VWF activity (%)
VWF antigen (%)
ADAMTS13 activity (%)
ADAMTS13 antigen (IU/ml)
CK-MB (U/L)
Troponin T (ng/L)
proBNP (pg/ml)
ICTP
SR Afib
ns
*
**
****
Sinus Rhythm
Afib
Patient char.
Neutrophil/NET
Inflammation
Thrombosis
Cardiac
Echo
Age
cCHADSVASC
Neu_abs
MPODNA
H3Cit
PAD4
IL6
TNFa
Fib
DD
TAT
AT
sPsel
VWF_act
VWF_Ag
ATS_Ag
ATS_Act
CKMB
Trop_T
proBNP
ICTP
LAA_flow
LAVI
EF
EE
Individuals - PCA
Dim1 (31.7%)
Dim2 (10.2%)
Afib
A
S

Figure S6

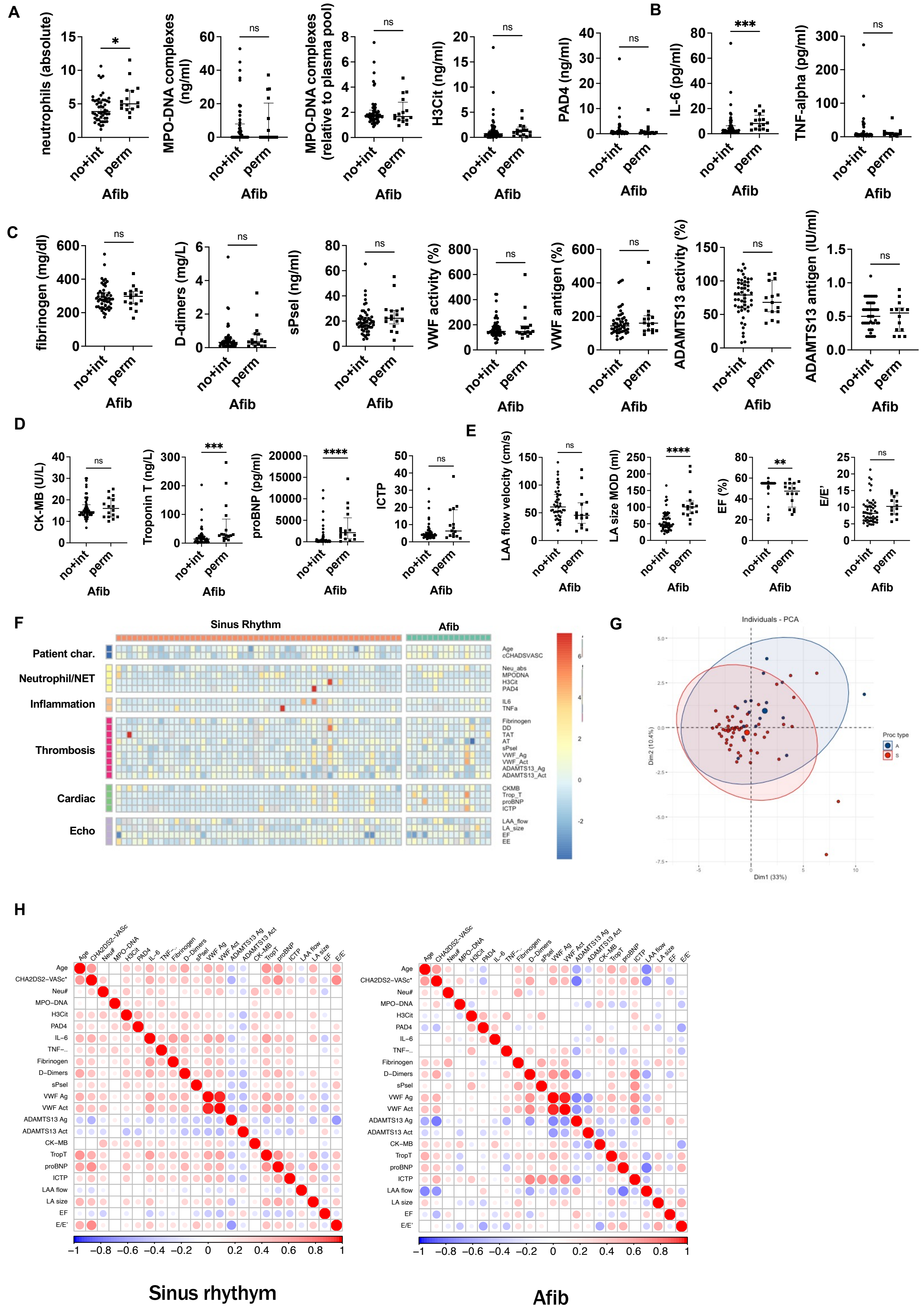
A
neutrophils (absolute)
MPO-DNA complexes (ng/ml)
MPO-DNA complexes (relative to plasma pool)
H3Cit (ng/ml)
PAD4 (ng/ml)
B
IL-6 (pg/ml)
TNF-alpha (pg/ml)
C
fibrinogen (mg/dl)
D-dimers (mg/L)
sPsel (ng/ml)
VWF activity (%)
VWF antigen (%)
ADAMTS13 activity (%)
ADAMTS13 antigen (IU/ml)
D
CK-MB (U/L)
Troponin T (ng/L)
proBNP (pg/ml)
ICTP
E
LAA flow velocity (cm/s)
LA size MOD (ml)
EF (%)
E/E'
no+int
perm
Afib
ns
*
**
***
****
F
Sinus Rhythm
Afib
Patient char.
Neutrophil/NET
Inflammation
Thrombosis
Cardiac
Echo
Age
cCHADSVASC
Neu_abs
MPODNA
H3Cit
PAD4
IL6
TNFa
Fibrinogen
DD
TAT
AT
sPsel
VWF_Ag
VWF_Act
ADAMTS13_Ag
ADAMTS13_Act
CKMB
Trop_T
proBNP
ICTP
LAA_flow
LA_size
EF
EE
G
Individuals - PCA
Dim1 (33%)
Dim2 (10.4%)
Proc type
A
S
H
Age
CHA2DS2-VASc*
Neu#
MPO-DNA
H3Cit
PAD4
IL-6
TNF-..
Fibrinogen
D-Dimers
sPsel
VWF Ag
VWF Act
ADAMTS13 Ag
ADAMTS13 Act
CK-MB
TropT
proBNP
ICTP
LAA flow
LA size
EF
E/E'
Sinus rhythm
Afib

Figure S7

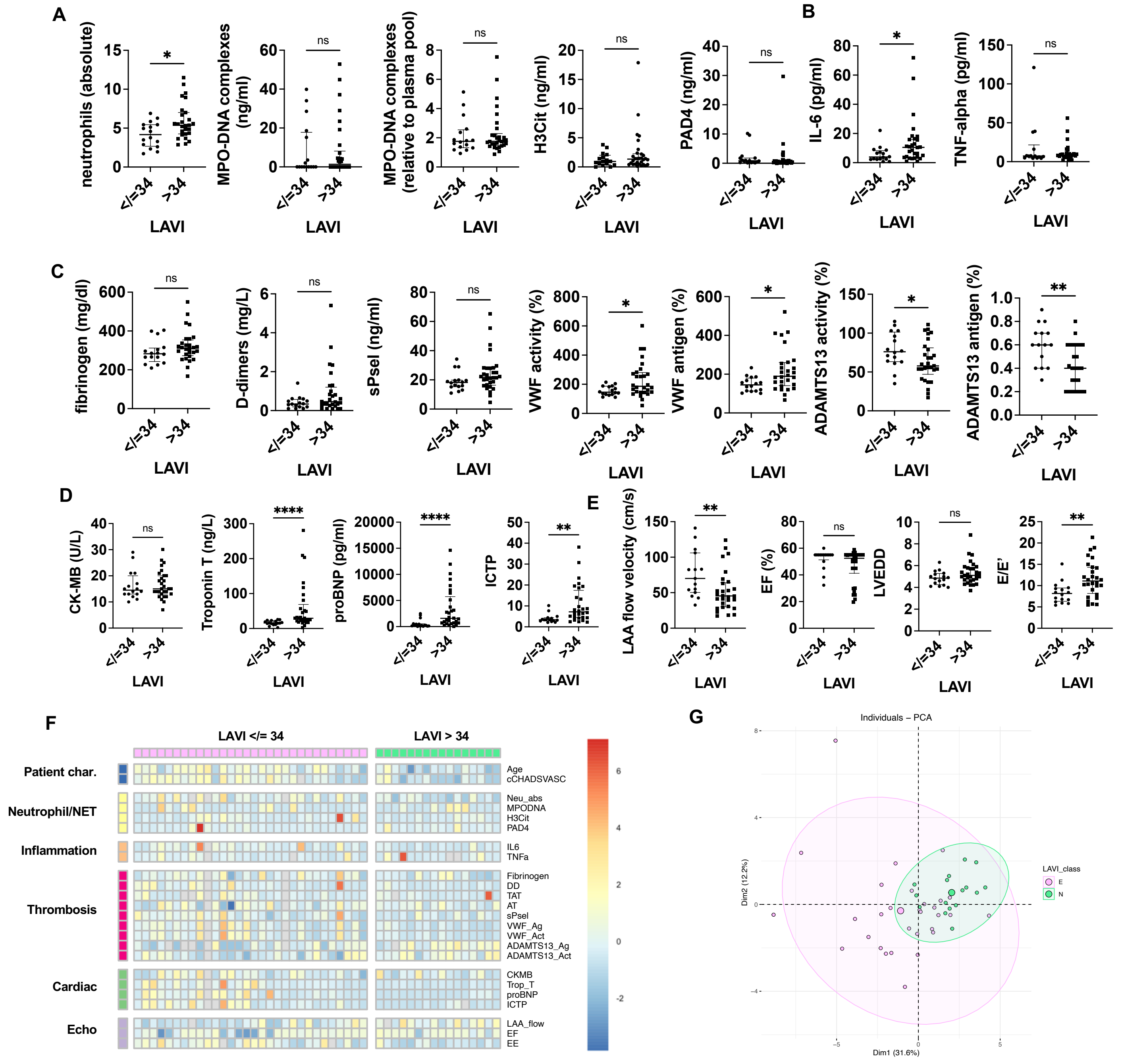

A
B
C
D
E
F
G
LAVI
ns
neutrophils (absolute)
MPO-DNA complexes (ng/ml)
MPO-DNA complexes (relative to plasma pool)
H3Cit (ng/ml)
PAD4 (ng/ml)
IL-6 (pg/ml)
TNF-alpha (pg/ml)
fibrinogen (mg/dl)
D-dimers (mg/L)
sPsel (ng/ml)
VWF activity (%)
VWF antigen (%)
ADAMTS13 activity (%)
ADAMTS13 antigen (%)
CK-MB (U/L)
Troponin T (ng/L)
proBNP (pg/ml)
ICTP
LAA flow velocity (cm/s)
EF (%)
LVEDD
E/E'
LAVI </= 34
LAVI > 34
Patient char.
Neutrophil/NET
Inflammation
Thrombosis
Cardiac
Echo
Age
cCHADSVASC
Neu_abs
MPODNA
H3Cit
PAD4
IL6
TNFa
Fibrinogen
DD
TAT
AT
sPsel
VWF_Ag
VWF_Act
ADAMTS13_Ag
ADAMTS13_Act
CKMB
Trop_T
proBNP
ICTP
LAA_flow
EF
EE
Individuals – PCA
Dim1 (31.6%)
Dim2 (12.2%)
LAVI_class
E
N

Figure S8

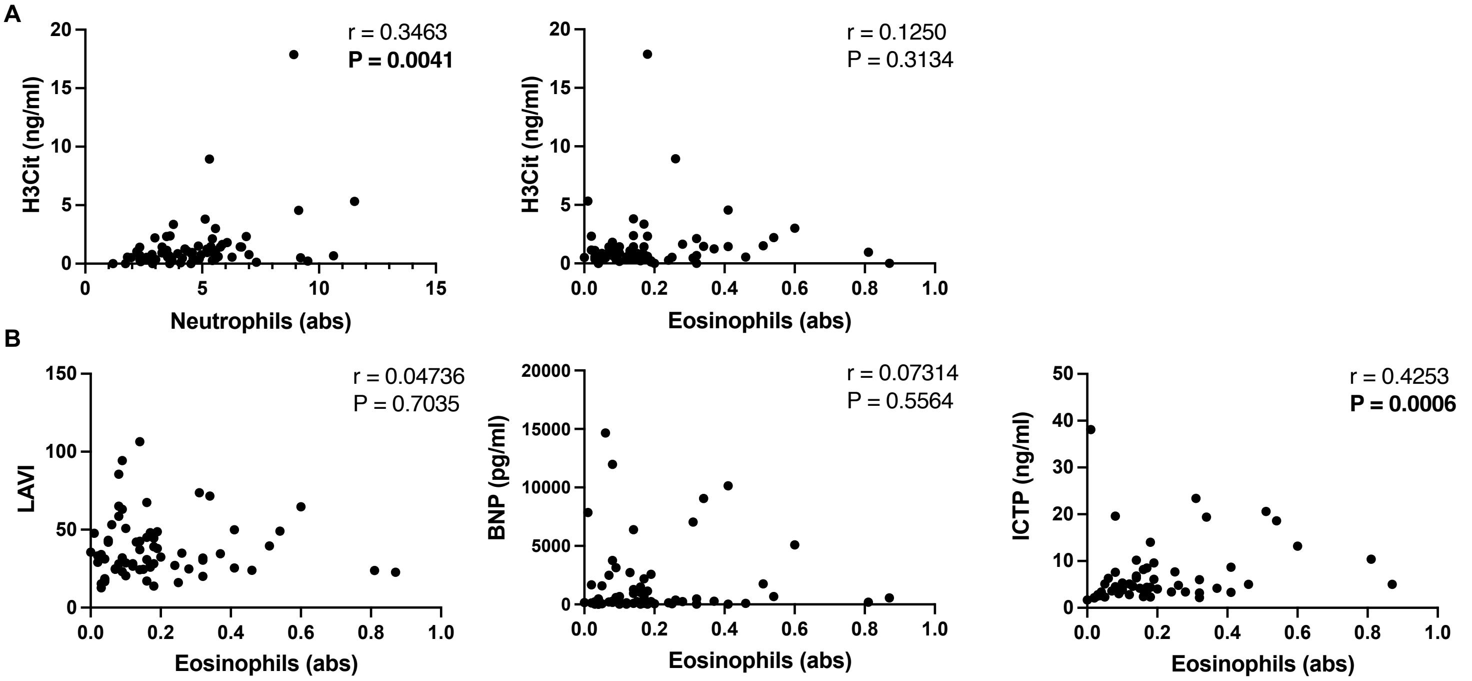
A
r = 0.3463
P = 0.0041
H3Cit (ng/ml)
Neutrophils (abs)
r = 0.1250
P = 0.3134
H3Cit (ng/ml)
Eosinophils (abs)
B
r = 0.04736
P = 0.7035
LAVI
Eosinophils (abs)
r = 0.07314
P = 0.5564
BNP (pg/ml)
Eosinophils (abs)
r = 0.4253
P = 0.0006
ICTP (ng/ml)
Eosinophils (abs)

Table S1

Coefficients[a]

Model		Unstandardized Coefficients		Standardized Coefficients			95.0% Confidence Interval for B		Correlations			Collinearity Statistics	
		B	Std. Error	Beta	t	Sig.	Lower Bound	Upper Bound	Zero-order	Partial	Part	Tolerance	VIF
1	(Constant)	-.780	1.944		-.401	.691	-4.719	3.160					
	XLN_Age	.910	.503	.277	1.811	.078	-.108	1.928	.319	.285	.252	.832	1.202
	XLN_neutrophils	.482	.188	.415	2.559	.015	.100	.863	.457	.388	.357	.737	1.357
	XLN_VWF_Ag	-.033	.189	-.030	-.176	.862	-.417	.350	.280	-.029	-.024	.661	1.514
	XLN_H3Cit	.021	.060	.053	.344	.733	-.101	.142	.218	.056	.048	.821	1.218

a. Dependent Variable: XLN_LAVI
